# Effects of intra-operative fluoroscopic 3D-imaging on peri-operative imaging strategy in calcaneal fracture surgery

**DOI:** 10.1007/s00402-017-2787-7

**Published:** 2017-09-21

**Authors:** M. S. H. Beerekamp, M. Backes, N. W. L. Schep, D. T. Ubbink, J. S. Luitse, T. Schepers, J. C. Goslings

**Affiliations:** 1 Trauma Unit, Department of Surgery, Academic Medical Center, 1105 AZ Amsterdam, The Netherlands; 2 Department of Surgery, Maasstad Hospital, Rotterdam, The Netherlands; 3 Department of Surgery, Academic Medical Center, Amsterdam, The Netherlands

**Keywords:** Calcaneus, Fracture, 3D-imaging, Intra-operative imaging, 2D-imaging

## Abstract

**Introduction:**

Previous studies demonstrated that intra-operative fluoroscopic 3D-imaging (3D-imaging) in calcaneal fracture surgery is promising to prevent revision surgery and save costs. However, these studies limited their focus to corrections performed after 3D-imaging, thereby neglecting corrections after intra-operative fluoroscopic 2D-imaging (2D-imaging). The aim of this study was to assess the effects of additional 3D-imaging on intra-operative corrections, peri-operative imaging used, and patient-relevant outcomes compared to 2D-imaging alone.

**Patients and methods:**

In this before–after study, data of adult patients who underwent open reduction and internal fixation (ORIF) of a calcaneal fracture between 2000 and 2014 in our level-I Trauma center were collected. 3D-imaging (BV Pulsera with 3D-RX, Philips Healthcare, Best, The Netherlands) was available as of 2007 at the surgeons’ discretion. Patient and fracture characteristics, peri-operative imaging, intra-operative corrections and patient-relevant outcomes were collected from the hospital databases. Patients in whom additional 3D-imaging was applied were compared to those undergoing 2D-imaging alone.

**Results:**

A total of 231 patients were included of whom 107 (46%) were operated with the use of 3D-imaging. No significant differences were found in baseline characteristics. The median duration of surgery was significantly longer when using 3D-imaging (2:08 vs. 1:54 h; *p* = 0.002). Corrections after additional 3D-imaging were performed in 53% of the patients. However, significantly fewer corrections were made after 2D-imaging when 3D-imaging was available (Risk difference (RD) −15%; 95% Confidence interval (CI) −29 to −2). Peri-operative imaging, besides intra-operative 3D-imaging, and patient-relevant outcomes were similar between groups.

**Conclusion:**

Intra-operative 3D-imaging provides additional information resulting in additional corrections. Moreover, 3D-imaging probably changed the surgeons’ attitude to rely more on 3D-imaging, hence a 15%-decrease of corrections performed after 2D-imaging when 3D imaging was available. No substantiation for cost reduction was found through reduction in peri-operative imaging or in terms of improved patient-relevant outcomes.

## Introduction

Restoration of anatomy to optimize functional outcome and lower the rate of secondary fusions is the main goal in calcaneal fracture surgery. Several research groups have described different pre-operative radiological fracture characteristics and measurements related to functional outcome [1–3]. Others have evaluated postoperative restoration of anatomy in relation to functional outcome [4, 5].

Intra-operative fluoroscopic 3D-imaging (3D-imaging), providing a reconstruction in slice images in the axial, coronal and sagittal planes as well as 3D volume rendering, in addition to conventional intra-operative fluoroscopic 2D-imaging (2D-imaging), may help evaluate the restoration of the anatomy and implant position. Since its introduction, more attention is given to the effect of the available intra-operative imaging modalities [6]. Most authors have focused solely on the number and type of corrections performed after additional 3D-imaging [7–15], suggesting that these additional corrections prevent revision surgery and reduce costs. However, little is known about the effect of the availability of intra-operative 3D-imaging on the surgeons’ attitude towards intra-operative 2D-imaging. In addition, little is known about the effects of intra-operative 3D-imaging on peri-operative imaging strategies, in terms of the evaluation of fracture characteristics, planning of the surgical procedure, postoperative evaluation of restoration of anatomy and implant position, and patient outcome [16].

Hence, the aim of this study was to assess the effects of intra-operative use of fluoroscopic 3D-imaging in patients with a calcaneal fracture on the number and type of intra-operative corrections of reduction and implant position, pre- intra- and postoperative (peri-operative) imaging used, and patient-relevant outcomes in terms of revision surgery, secondary fusions and infectious complications.

## Methods

In this before–after study, data of all patients with open reduction and internal fixation (ORIF) of a displaced intra-articular calcaneal fracture admitted to our academic level-1 trauma center from January 2000 until June 2014 were retrospectively collected. Potential eligible patients were detected with the corresponding operative procedure code. Patients were eligible for this study when ORIF was performed with the aim to restore anatomy. Patients younger than 18 years of age and patients with primary arthrodesis, revision of ORIF performed elsewhere and patients participating in a randomized trial (the EF3X-trial) that influenced the imaging strategy were excluded [17]. Intra-operative fluoroscopic 3D-imaging was clinically available in our hospital in 2007 and applied intra-operatively at the surgeons’ discretion and the availability of the 3D-C-arm.

Patient and fracture characteristics were collected from the hospitals electronic databases. These included age, gender, Body Mass Index (BMI), American Society of Anesthesiologists (ASA) classification and relevant risk factors like diabetes mellitus and smoking. Other variables were the trauma mechanism, Injury Severity Score (ISS), fracture side, presence of an open fracture, bilateral fracture and presence of a fracture of the ipsilateral foot/ankle. Calcaneal fractures were classified according to the Sanders classification [18]. The time between the fracture and surgery was recorded and expressed in days.

Types of pre- and postoperative radiological exams of the calcaneus were extracted from the patients’ radiology charts.

Surgery reports were reviewed to determine duration of surgery, type of intra-operative imaging used and the number and type of surgical corrections performed during the same procedure following 2D- and 3D-fluoroscopic imaging. Both 2D- and 3D-imaging could be used at the surgeons’ discretion at any time during surgery when 3D-imaging was available. Corrections were defined as a description of a revision of the reduction (for example intra-articular step-off, gap or tuber position) or a revision of the fixation (for example plate position or screw length or position). In case the implants had to be removed to correct the reduction, this was only counted as a correction in reduction. Patient outcomes, defined as the number of revision operations, wound complication rates, implant removal and number of secondary arthrodeses were determined by reviewing the patient chart until October 2015, resulting in a minimum follow-up duration of 15 months.

### Intra-operative fluoroscopic imaging

**Fig. 1:**
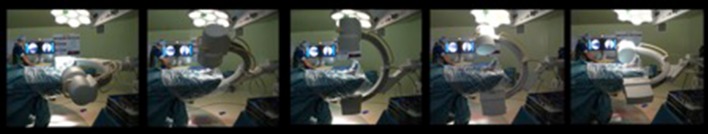
Rotation of the 3D-RX-system; for a single 3D scan with the BV Pulsera (Philips Medical Systems, Best, The Netherlands) a series of 225 2D fluoroscopic images is acquired over a period of 30 s during a motorized 200° rotation of the C-arm. The projection images are used to reconstruct a 3D dataset

**Fig. 2:**
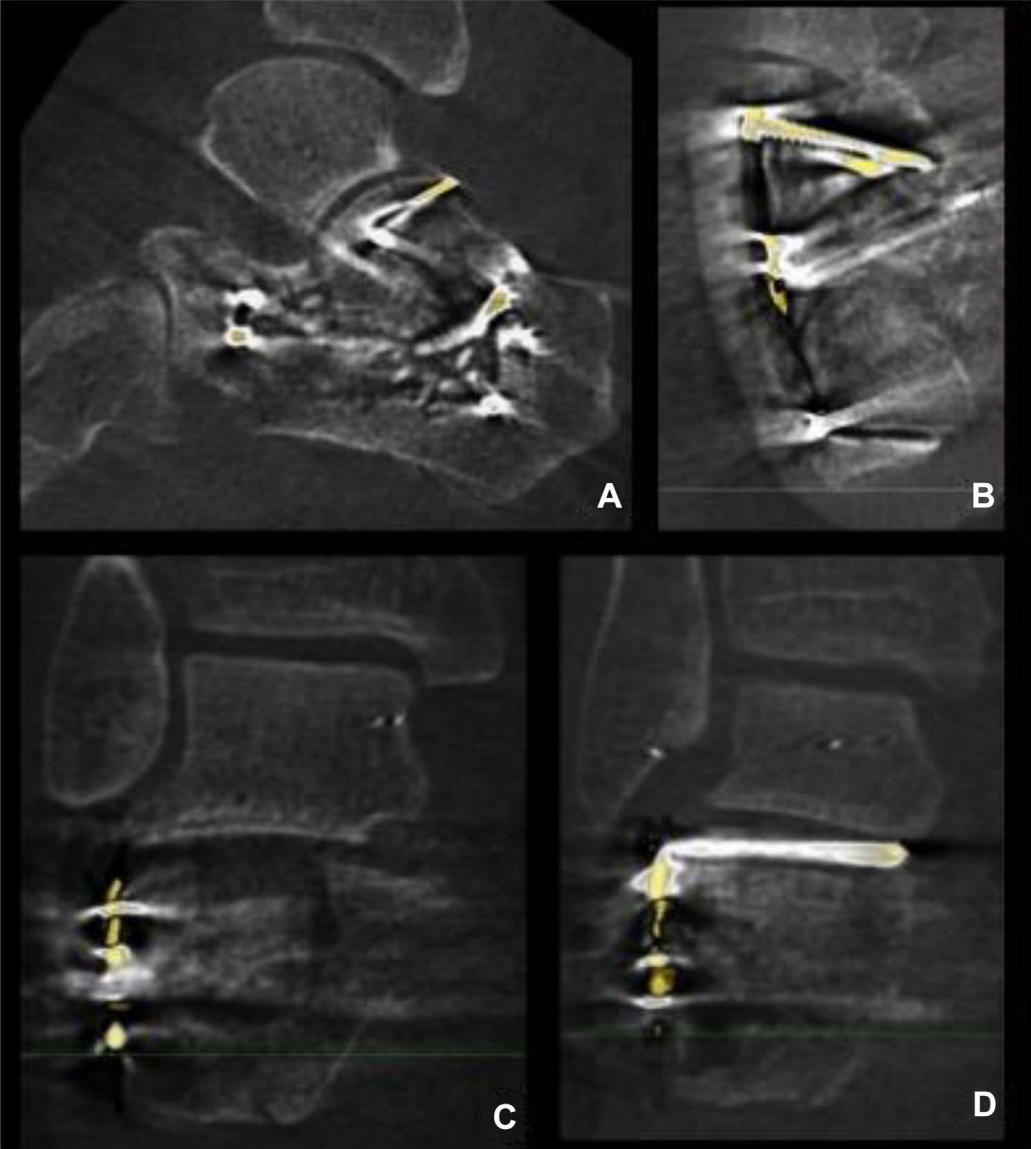
3D-images of intra-articular step, gap and implant position of the calcaneus; Sagital (**a**), axial (**b**) and coronal slice images (**c**, **d**) of intra-operative fluoroscopic 3D-imaging. Titanview software is used to color the metal implants present (Titanview software, Philips Healthcare, Best, the Netherlands). **a** Step-off in calcanocuboid (CC) joint. **b** Gap in calcanocuboid (CC) joint. **c** Step-off in posterior talocalcaneal (PTC) joint. **d** Intra-articular position of a screw in the posterior talocalcaneal (PTC) joint

Both intra-operative fluoroscopic 2D and 3D-imaging were performed with the BV Pulsera with 3D-RX (3 Dimensional Rotational X-ray) [9]. The BV Pulsera (Philips Healthcare, Best, the Netherlands) consists of a mobile C-arm unit modified to provide a motorized rotational movement and is combined with a Philips 3D-RA workstation. For a single 3D scan, a series of 225 2D fluoroscopic images is acquired over a period of 30 s during a 200° rotation of the C-arm (Fig. 1). The projection images are used to reconstruct a 3D data set. Both volume-rendering and slice images in the axial, coronal and sagittal planes were available. The slice images were considered to provide the best information and were used solely in clinical practice (Fig. 2). From autumn 2005, 3D-imaging of the BV Pulsera was used in a research setting; from 2007, the BV Pulsera was clinically available. From 2009, the images could be enhanced by coloring the metal implants present (Titanview software, Philips Healthcare, Best, the Netherlands).

The radiation exposure of each image in the scanning run is dynamically adjusted to provide the best combination of low dose and optimal image quality. The maximum equivalent dosage of a 3D scan of the calcaneus with the BV Pulsera is 17 mSv. Because 3D-imaging is more time-consuming and requires additional preparation to remain sterility, 3D-imaging was used additional to 2D-imaging at the surgeon’s preference.

### Statistical analysis

Descriptive statistics were applied to analyze baseline and peri-operative characteristics and patient outcomes using the Statistical Package for the Social Sciences (SPSS version 23, IBM, Armonk, NY, USA) and Openepi (version 3.01, online resource) [19]. Patients were divided into two groups depending on whether or not intra-operative fluoroscopic 3D-imaging was conducted (No-3D group, 3D group). Continuous data with a normal distribution were expressed as means with standard deviations. Mean differences with their 95% confidence intervals were calculated. Non-normally distributed data were expressed as medians with their range and tested with the Mann–Whitney *U* test. A *p* value of <0.05 was considered significant. Proportional data of the categorical data were given and expressed as risk differences and risk ratios, both with 95% confidence intervals.

## Results

### Patient inclusion

**Fig. 3:**
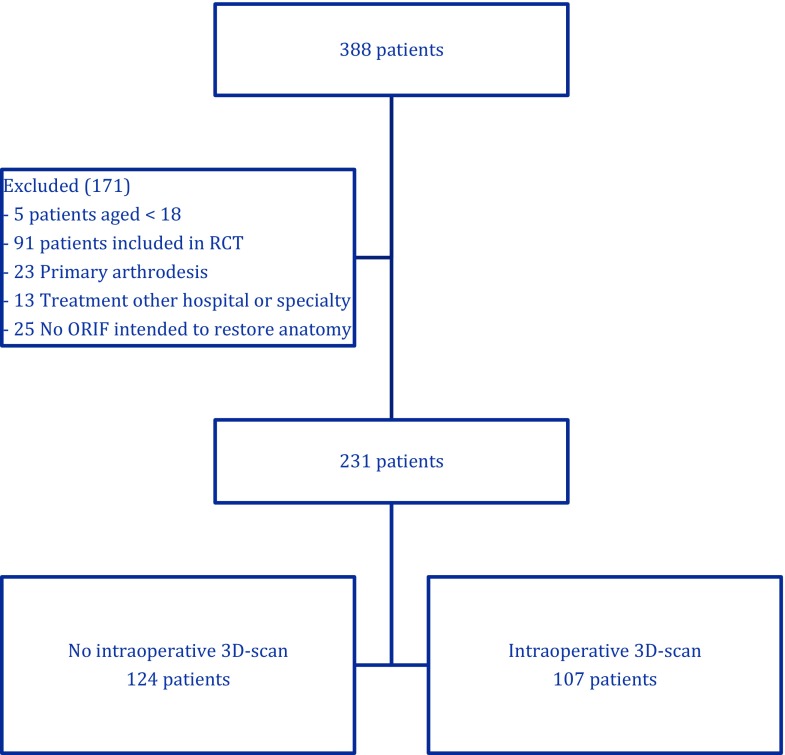
Flowchart of patient inclusion

During the inclusion period, 388 patients were identified (Fig. 3), of whom 171 were excluded because of their age (<18 years), a primary arthrodesis, participation in another trial or because of a previous ORIF of the calcaneus elsewhere. In 107 of the 231 included patients (46%) intra-operative 3D-imaging was used.

### Baseline characteristics

No differences were found in baseline characteristics between the two patient groups (Table 1). Patients in the no-3D and 3D groups had a mean age of 43 versus 45 years, respectively [mean difference 1.87 (95% CI −4.4 to 3.0)]. No significant differences were found in age, gender, ASA-classification and relevant risk factors like diabetes or smoking. Most patients had fallen from a height (70 vs. 60%) or from the stairs (27 vs. 26%). Eleven percent of patients in the No-3D group had an ISS > 16, compared to 7% in the 3D group (RD −4.7%, 95% CI −12.0 to 2.5). In the 3D-group, the duration of surgery was significantly longer with a median time of 2:08 h (range 1:06–8:44) compared to 1:54 h (range 0:52–6:45) (*p* = 0.002) in the no-3D group.

**Table 1:** Patient, fracture and operation characteristics

Characteristic	No-3D *n* (%)	3D *n* (%)	Mean diff. [95% CI]	Risk difference % [95% CI]	Risk ratio [95% CI]	*p*-value
Gender male	73 (59)	72 (67)		8.4 [−4.0 to 20.8]	1.1 [0.9 to 1.4]	
Age (mean)	43 (41–45)	45 (42–47)	1.87 [−4.4 to 3.0]			0.723
Body mass index (BMI)				−1.3 [−4.1 to 1.5]	1.0 [0.8 to 1.3]	
<18.5	7 (6)	2 (2)		−4.1 [−9.3 to 1.1]	0.3 [0.1 to 1.5]	
18.5 – 25	64 (56)	55 (56)		−0.6 [−14.0 to 12.8]	1.0 [0.8 to 1.3]	
25–30	28 (25)	35 (35)		10.8 [−1.5 to 23.1]	1.4 [0.9 to 2.2]	
30–35	13 (11)	4 (4)		**−7.4 [−14.4 to −0.3]**	**0.4 [0.1 to 1.1]**	
>35	2 (2)	3 (3)		1.3 [−2.9 to 5.4]	1.7 [0.3 to 10.1]	
ASA				−1.5 [−5.3 to 2.4]	1.0 [0.8 to 1.1]	
1	86 (69)	69 (65)		−4.9 [−17.0 to 7.3]	0.9 [0.8 to 1.1]	
2	33 (27)	36 (34)		7.0 [−4.8 to 18.9]	1.3 [0.9 to 1.9]	
3	5 (4)	2 (2)		−2.2 [−6.5 to 2.1]	0.5 [0.1 to 2.3]	
Diabetes Mellitus	7 (6)	6 (6)		−0.0 [−6.0 to 5.9]	1.0 [0.3 to 2.9]	
Smoking	65 (54)	48 (47)		−7.1 [−20.2 to 6.0]	0.9 [0.7 to 1.1]	
Trauma mechanism				0.9 [−1.0 to 2.8]	1.0 [0.8 to 1.2]	
Fall from height	87 (70)	64 (60)		−9.8 [−22.1 to 2.5]	0.9 [0.7 to 1.0]	
Fall from stairs	17 (14)	27 (26)		**11.8 [1.5 to 22.0]**	**1.9 [1.1 to 3.2]**	
Car accident	8 (7)	2 (2)		−4.6 [−9.6 to 0.5]	0.3 [0.1 to 1.3]	
Motor accident	0 (0)	2 (2)		1.9 [−0.7 to 4.5]	–	
Direct trauma	2 (2)	5 (5)		3.1 [−1.5 to 7.7]	2.9 [0.6 to 14.8]	
Other	10 (8)	6 (6)		−2.4 [−8.9 to 4.1]	0.7 [0.3 to 1.9]	
ISS ≥ 16	14 (11)	7 (7)		−4.7 [−12.0 to 2.5]	0.6 [0.2 to 1.4]	
Left-side fracture	62 (50)	42 (39)		−10.8 [−23.5 to 2.0]	0.8 [0.6 to 1.1]	
Open fracture	2 (2)	3 (3)		1.2 [−2.7 to 5.0]	1.7 [0.3 to 10.6]	
Bilateral fracture	23 (19)	15 (14)		−4.5 [−14.0 to 5.0]	0.8 [0.4 to 1.4]	
Fracture ipsilateral foot or ankle	13 (11)	13 (12)		1.7 [−6.5 to 9.8]	1.2 [0.6 to 2.4]	
Sanders fracture type				0.7 [−2.7 to 4.1]	1.0 [0.8 to 1.2]	
1	9 (8)	9 (9)		0.8 [−6.9 to 8.5]	1.1 [0.5 to 2.6]	
2	73 (68)	67 (68)		0.1 [−12.7 to 12.8]	1.0 [0.8 to 1.2]	
3	24 (22)	20 (20)		−2.0 [−13.2 to 9.1]	0.9 [0.5 to 1.5]	
4	2 (2)	3 (3)		1.2 [−3.0 to 5.4]	1.6 [0.3 to 9.6]	
Days to surgery, median (range)	13.0 (1–24)	15.0 (2–60)				0.060
Duration of surgery, median (range)	1:54 (0:52–6:45)	2:08 (1:06–8:44)				0.002

Bold values indicate a significant difference between the groups

*CI* confidence interval

### Peri-operative imaging and intra-operative corrections

Almost every patient underwent a pre-operative CT scan (100 vs. 98%) (Table 2). In the 3D group, a pre-operative MRI scan was obtained in one patient. An Anterior–Posterior (AP) and a lateral view were also obtained in almost all patients (99 vs. 97%), in contrast axial views, were obtained in only 53 vs. 52% of the patients. Broden’s views were performed more often in the No-3D group (34%) than in the 3D-group (20%) (RD 13.9%, 95% CI −25.9 to −1.8).

**Table 2:** Peri-operative imaging and intra-operative corrections

	No-3D *n* (%)	3D *n* (%)	Risk difference % [95% CI]	Risk ratio [95% CI]
Preoperative imaging			0.6 [−0.9 to 2.1]	1.0 [0.8 to 1.2]
X-ray	1 (1)	1 (1)	0.1 [−2.4 to 2.6]	1.1 [0.1 to 17.8]
CT scan	45 (39)	47 (45)	6.7 [−6.2 to 19.7]	1.2 [0.9 to 1.6]
X-ray & CT scan	71 (61)	55 (53)	−7.8 [−20.9 to 5.3]	0.9 [0.7 to 1.1]
Other	0 (0)	1 (1)	1.0 [−0.9 to 2.8]	–
Type of preoperative X-ray				
AP & lateral	92 (99)	85 (97)	−2.3 [−6.7 to 2.0]	1.0 [0.9 to 1.0]
Axial	55 (53)	50 (52)	−0.8 [−14.7 to 13.0]	1.0 [0.8 to 1.3]
Broden	35 (34)	19 (20)	−**13.9 [**−**25.9 to** −**1.8]**	**0.6 [0.4 to 1.0]**
Overall corrections performed	61 (53)	70 (69)	**16.3 [3.5 to 29.1]**	**1.3 [1.1 to 1.6]**
Corrections performed after 2D-imaging	61 (53)	38 (38)	−**15.4 [**−**28.6 to** −**2.3]**	**0.7 [0.5 to 1.0]**
Number of corrections after 2D-imaging			−3.4 [−6.6 to −0.3]	1.0 [0.8 to 1.2]
0	54 (47)	63 (62)	**15.4 [2.3 to 28.55]**	**1.3 [1.0 to 1.7]**
1	39 (34)	29 (29)	−5.2 [−17.6 to 7.2]	0.8 [0.6 to 1.3]
2	17 (15)	9 (9)	−5.9 [−14.4 to 2.7]	0.6 [0.3 to 1.3]
3	5 (4)	0 (0)	−4.3 [−8.1 to −0.6]	–
Type of correction after 2D-imaging			−3.5 [−21.0 to 14.0]	0.9 [0.7 to 1.3]
Reduction	39 (43)	22 (47)		
Implant position	51 (57)	25 (53)		
Year 3D-imaging performed			54.0 [43.6–64.5]	5.0 [2.9–8.7]
Before 2007	78 (87)	12 (13)		
After 2007	46 (33)	95 (67)		
Number of 3D scans				
1	–	90 (84)		
2	–	16 (15)		
3	–	1 (1)		
Number of corrections after 3D-imaging				
0	–	48 (47)		
1	–	36 (35)		
2	–	13 (13)		
3		4 (4)		
4		0 (0)		
5	–	1 (1)		
Timing 3D scan				
Before reduction & hardware implantation	–	0 (0)		
After reduction	–	12 (10)		
After reduction & hardware implantation	–	113 (90)		
Type of correction after 3D-imaging				
Reduction	–	2 (4)		
Implant position	–	51 (96)		
Postoperative imaging				
X-ray	118 (98)	102 (95)	−2.2 [−7.1 to 2.7]	1.0 [0.9 to 1.0]
CT scan	0 (0)	0 (0)	–	–
X-ray & CT scan	3 (3)	5 (5)	2.2 [−2.7 to 7.0]	1.9 [0.5 to 7.7]
Other	0 (0)	0 (0)	–	–
Type of postoperative X-ray				
AP & lateral	118 (100)	107 (100)	–	–
Axial	89 (75)	86 (80)	5.0 [−5.9 to 15.8]	1.1 [0.9 to 1.2]
Broden	37 (31)	37 (35)	3.2 [−9.1 to 15.5]	1.1 [0.8 to 1.6]

Bold values indicate a significant difference between the groups

*CI* confidence interval

Intra-operative 2D-imaging was used during all operations. One or more corrections after intra-operative imaging were performed in 53% of the operations in the No-3D group versus 69% in the 3D-group. Significantly less corrections were made after 2D-imaging in the 3D-group (38%) compared to the No-3D group (53%); RD −15.4%, 95% CI −28.6 to −2. In both groups, usually only one correction was performed, with slightly more corrections in implant position (53 vs. 57%) than reduction (43 vs. 47%).

In 107 procedures, additional 3D-imaging was performed; most often once (84%), but sometimes two or three times during the surgical procedure. Most procedures with 3D-imaging were performed from the beginning of the year 2007. After 2007, a 3D scan was performed in 67% of the surgical procedures. Ninety percent of the scans were obtained after reduction and fixation, while the remaining 10% was performed after fracture reduction but before definitive hardware implantation. When 3D-imaging was available, in more than half (53%) of the operations an additional correction was performed following 3D-imaging. In contrast to corrections following 2D-imaging, 96.2% of the corrections were corrections of implant (plate and/or screw) position.

All patients underwent postoperative X-ray imaging and, in 3% and 5% respectively, a postoperative CT scan was performed. In contrast with preoperative imaging, in both groups Broden’s views were taken in approximately one-third of the patients. AP and lateral views were obtained in all patients and axial views in 75–80% of them.

### Outcomes

No significant differences were found in patient outcomes between the two groups (Table 3). Revision surgery was deemed necessary in 2% versus 3% of patients following ORIF. Wound infections occurred in 25% versus 33% of patients of which the majority was superficial. Implants were removed in less than half of the patients, mainly due to painful symptoms. Secondary arthrodeses were performed in 7% of patients in the no-3D group and 11% in the 3D-group, mainly due to a painful joint.

**Table 3:** Patient-relevant outcomes

	No-3D *n* (%)	3D *n* (%)	Risk difference % [95% CI]	Risk ratio [95% CI]
Revision surgery	2 (2)	3 (3)	1.2 [−2.6 to 5.0]	1.7 [0.3 to 10.2]
Wound infection	31 (25)	35 (33)	7.5 [−4.2 to 19.3]	1.3 [0.9 to 2.0]
Type of wound infection			2.9 [−3.1 to 8.9]	1.0 [0.7 to 1.5]
Superficial without antibiotics	7 (23)	7 (20)	−2.6 [−22.4 to 17.2]	0.9 [0.3 to 2.2]
Superficial with antibiotics	13 (42)	15 (43)	0.9 [−23.0 to 24.8]	1.0 [0.6 to 1.8]
Deep with debridement	6 (19)	6 (17)	−2.2 [−20.9 to 16.5]	0.9 [0.3 to 2.5]
Deep with hardware removal	5 (16)	5 (14)	−1.8 [−19.2 to 15.5]	0.9 [0.3 to 2.8]
Osteomyelitis	0 (0)	2 (6)	5.7 [−2.0 to 13.4]	–
Implant removal	58 (47)	45 (42)	−4.7 [−17.6 to 8.1]	0.9 [0.7 to 1.2]
Reason for implant removal			−0.3 [−5.6 to 5.0]	1.0 [0.8 to 1.3]
Pain	43 (75)	31 (69)	−6.6 [−24.1 to 11.0]	0.9 [0.7 to 1.2]
Material related	3 (5)	6 (13)	8.1 [−3.4 to 19.6]	2.5 [0.7 to 9.6]
Infection	8 (14)	7 (16)	1.5 [−12.4 to 15.4]	1.1 [0.4 to 2.8]
Planned removal	3 (5)	1 (2)	−3.0 [−10.3 to 4.2]	0.4 [0.0 to 3.9]
Arthrodesis	8 (7)	11 (11)	4.0 [−3.2 to 11.3]	1.6 [0.7 to 3.9]
Reason for arthrodesis				
Pain	6 (75)	7 (78)	2.8 [−37.7 to 43.3]	1.0 [0.6 to 1.8]
Persisting infection	2 (25)	2 (22)	−2.8 [−43.3 to 37.7]	0.9 [0.2 to 4.9]

*CI* confidence interval

## Discussion

In this study, we found that when 3D-imaging is available at the surgeon’s preference additional corrections were performed in 53% of the patients, which were not performed after 2D-imaging. In addition, when the surgeon has 3D-imaging at his disposal the number of corrections performed after 2D-imaging decreases with 15%. These additional corrections are probably conducted because the increased information 3D-imaging gives about fracture reduction and implant position. However, the reduction in corrections performed after 2D-imaging also suggests that the surgeons’ attitude towards 2D-imaging changes unwittingly when 3D-imaging is also available: they tend to rely more on 3D-imaging and postpone their decision to correct until 3D-imaging has been performed.

Additionally, following 3D-imaging most of the corrections were performed because of a suboptimal implant position, while after 2D-imaging corrections of both reduction and implant position were performed. A reason for this difference could be that reduction can be evaluated adequately with 2D-imaging, while implant position is more difficult to evaluate based on these images. Another explanation could be the timing of 3D-imaging, which is most often at the end of the procedure. The threshold to optimize reduction at this stage of the procedure could be higher, because mostly different implants need to be removed. The threshold to revise only one or more screws because of length or position is lower and could therefore be done more frequently. The number of corrections in our study is slightly higher than the approximately 40% correction rate found in previous studies [12, 20]. However, the higher correction rate of implant position as found in the present study was also shown by others [15, 20].

No differences were found in the patient-relevant outcomes or peri-operative imaging, except for the presence of pre-operative Broden’s views. This difference might be due to the nationwide trend towards centralization for complex fracture care, in which our hospital became a referral center for calcaneal fractures. In the referring hospitals less Broden’s views could have been performed.

As described in the literature, 3D-imaging shows a better sensitivity for the evaluation of both reduction and implant position (ranging from 76–100%) than 2D-imaging (63–95%) and its results are similar to computer tomography [21–23]. There is not yet literature known describing the diagnostic accuracy specific for the BV Pulsera. However, in our study no reduction in number and type of pre-, intra- and postoperative radiological exams was found when 3D-imaging was available. Various authors have suggested that corrections performed after intra-operative 3D-imaging can reduce the number of revision surgery [7, 8, 10, 12, 20, 24]. However, we found no difference in the need for revision surgery. An explanation for this could be overestimation of the number of additional corrections after 3D-imaging, because of a more critical attitude of the surgeon towards 2D-imaging when 3D-imaging is not available. Another explanation may be that the disadvantages of a reoperation outweigh the expected advantages of correcting a suboptimal reduction and/or implant position: the threshold for a reoperation is high. No differences were found in the percentage of patients requiring implant removal or secondary arthrodesis, indicating that these are legitimate considerations.

This was a before–after study comparing the effect of additional 3D-imaging groups on peri-operative imaging and patient outcome. After the clinical introduction of 3D-fluoroscopic imaging in 2007, the application of 3D-imaging was at the surgeons’ discretion, which was done in 67% of the operations. Although reasons for not using 3D-imaging could not be retrieved retrospectively, unavailability of the 3D-C-arm due to maintenance or repair was likely to be the main reason. However, selection bias cannot be excluded. Additionally, the software enhanced with Titanview during the study period, which could have improved the diagnostic accuracy.

In addition, the retrospective character of this study could have led to underestimation of the number and type of corrections performed, especially after 2D-imaging. In contrast to 2D-imaging, the surgeon has to make preparations for 3D-imaging in order to preserve sterility in the operation area and is, therefore, more conscious of the corrections made and more likely to report these corrections in the operation chart than when he uses 2D imaging more continuously during the procedure. However, we expect that this underestimation is similar in both groups, because the use of 2D-imaging is the same.

Our findings suggest no differences in patient outcome in terms of wound complications, revision surgery, or hardware removal. In addition, Gwak et al. did not find differences in the AOFAS hindfoot score or Visual Analog Scale [13]. Unfortunately, no patient-reported outcome measures were taken into account in our study.

Follow-up of a multicenter randomized trial is ongoing to answer the question whether the use of additional 3D-imaging improves the quality of reduction and fixation and patient outcomes [17]. In this study, the availability of intra-operative 3D-imaging is determined by randomization, not until the surgeon is satisfied about the reduction and fixation based on fluoroscopic 2D-imaging and is ready to end the operation. Radiologic outcome is determined as well as functional outcome by patient-rated outcome measures. The results of this trial are expected in the summer of 2017.

## Conclusions

The intra-operative availability of 3D-imaging during fracture surgery of the calcaneus leads to additional corrections in 53%. Moreover, 3D-imaging changed the surgeons’ attitude to rely more on 3D-imaging, hence a 15%-decrease of corrections performed after 2D-imaging when 3D imaging was available.

In addition, in our study no differences in peri-operative imaging and patient-relevant outcomes are found. Therefore, previous conclusions that corrections performed after intra-operative 3D-imaging are always additional corrections and may reduce revision surgery and costs require better underpinning.
